# Functional Analysis of the *PgCesA3* White Spruce Cellulose Synthase Gene Promoter in Secondary Xylem

**DOI:** 10.3389/fpls.2019.00626

**Published:** 2019-05-28

**Authors:** Juliana Stival Sena, Denis Lachance, Isabelle Duval, Thi Thuy An Nguyen, Don Stewart, John Mackay, Armand Séguin

**Affiliations:** ^1^Department of Wood and Forest Sciences, Université Laval, Quebec City, QC, Canada; ^2^Natural Resources Canada, Canadian Forest Service, Laurentian Forestry Centre, Quebec City, QC, Canada; ^3^Department of Plant Sciences, University of Oxford, Oxford, United Kingdom

**Keywords:** conifer, cellulose synthase phylogeny, plant genetic transformation, functional analysis, electrophoretic mobility shift assay, MYB regulatory binding sites

## Abstract

Cellulose is an essential structural component of the plant cell wall. Its biosynthesis involves genes encoding cellulose synthase enzymes and a complex transcriptional regulatory network. Three cellulose synthases have been identified in conifers as being potentially involved in secondary cell wall biosynthesis because of their preferential expression in xylem tissues; however, no direct functional association has been made to date. In the present work, we characterized the white spruce [*Picea glauca* (Moench) Voss] cellulose synthase *PgCesA3* gene and 5′ regulatory elements. Phylogenetic analysis showed that *PgCesA1-3* genes grouped with secondary cell wall-associated Arabidopsis cellulose synthase genes, such as *AtCesA8, AtCesA4*, and *AtCesA7*. We produced transgenic spruce expressing the GUS reporter gene driven by the *PgCesA3* promoter. We observed blue staining in differentiating xylem cells from stem and roots, and in foliar guard cells indicating that *PgCesA3* is clearly involved in secondary cell wall biosynthesis. The promoter region sequence of *PgCesA3* contained several putative MYB cis-regulatory elements including AC-I like motifs and secondary wall MYB-responsive element (SMRE); however, it lacked SMRE4, 7 and 8 that correspond to the sequences of AC-I, II, and III. Based on these findings and results of previous transient trans-activation assays that identified interactions between the *PgCesA3* promoter and different MYB transcription factors, we performed electrophoretic mobility shift assays with MYB recombinant proteins and cis-regulatory elements present in the *PgCesA3* promoter. We found that PgMYB12 bound to a canonical AC-I element identified in the *Pinus taeda* PAL promoter and two AC-I like elements. We hypothesized that the PgMYB12 could regulate *PgCesA3* in roots based on previous expression results. This functional study of *PgCesA3* sequences and promoter opens the door for future studies on the interaction between PgMYBs and the *PgCesA3* regulatory elements.

## Introduction

Cellulose is a fundamental constituent of the plant cell wall. It represents half of the mass of wood and is a crucial resource for the production of pulp, biomaterials and biofuels (Keijsers et al., 2013). Plants and trees accumulate large amounts of crystalline cellulose during the formation of secondary cell walls, which provide mechanical strength and are the main hallmark of secondary xylem and woody tissues. A protein complex formed of cellulose synthase enzymes that are specific to the secondary cell wall is responsible for building the cellulose microfibrils that make up the extracellular matrix (Zhong and Ye, 2015). In conifers, three cellulose synthases have been identified that are putatively involved in secondary cell wall formation but no direct functional association has been made to date. The regulation of cellulose synthase genes remains only partially understood in plants and trees in spite of their central role in development.

Several of the secondary cell wall cellulose synthases have been identified in angiosperm plants and their molecular function has been the subject of several reports. In *Arabidopsis thaliana*, there are ten celluloses synthase genes, three of which make up the cellulose synthase complex for secondary wall deposition (Endler and Persson, 2011). A study using specific antibodies to the three catalytic subunits of the cellulose synthase complex (CSC) and a green fluorescent protein fusion indicated that all three subunits are required to assemble a functional complex in the developing xylem in Arabidopsis (Gardiner et al., 2003). The CSC is composed of a hexamer of catalytically active CesA trimmers, with each of CesA in equimolar amounts (Hill et al., 2014). Mutations in any of these three genes caused a reduction of cellulose content and secondary wall thickening, and a collapsed xylem phenotype (Taylor et al., 2003; Zhong and Ye, 2015). The same phenotype was observed in *CesA* mutants from other vascular plants such as rice and poplar (Tanaka et al., 2003; Joshi et al., 2011; Handakumbura et al., 2013).

In plants, other than model species there is a growing interest in understanding the role of cellulose synthases. Recent studies have deciphered the expression patterns of cellulose synthases in tobacco (Xu and Kong, 2017), flax (Galinousky et al., 2017), and soybean (Nawaz et al., 2017). In conifers, *CesA* genes have been identified in large-scale gene expression studies (Raherison et al., 2012; Jokipii-Lukkari et al., 2017). Three cellulose synthase genes in white spruce and loblolly pine (*CesA1, CesA2, CesA3*) were identified as being potentially involved in secondary cell wall formation based on their high expression in secondary xylem and sequence similarity to angiosperm *CesA* genes (Nairn and Haselkorn, 2005; Raherison et al., 2012). However, direct functional association with secondary cell wall formation is lacking and their precise expression patterns remain to be tested through *in situ* hybridization, promoter/reporter studies or other techniques.

A network of transcription factors regulates secondary cell wall biosynthesis in plants. Closely related NAC domain transcription factors activate several downstream transcription factor genes that directly trigger secondary cell wall biosynthesis genes (Zhong et al., 2010). Many of the downstream transcriptional regulators are R2R3-MYB transcription factors (Zhong et al., 2010), including AtMYB46, AtMYB52, AtMYB54, AtMYB58, AtMYB63, AtMYB83, AtMYB85, and AtMYB103 (Zhong and Ye, 2014). In conifers, molecular switches that regulate secondary cell wall were identified. For example, the *Pinus pinaster* transcription factor *PpNAC1*, a functional ortholog of the Arabidopsis *SND1* and *NST1*, activated the R2R3-MYB transcription factor *PpMYB4*, which activated *PpMYB8* (Pascual et al., 2018). In white spruce, Duval et al. (2014) identified the *PgNAC7* that could play a master regulatory role and activated *PgMYB8*, which activated *PgMYB1*. They also have reported a positive interaction of several MYB transcription factors with a white spruce *CesA3* promoter. The transcription factors that trans-activated the spruce *CesA3* promoter included *Picea glauca* PgMYB11, PgMYB12, and PgMYB22, and *Pinus taeda* PtMYB1 and PtMYB8 (Duval et al., 2014). The *Pt/PgMYB8* genes were implicated in the regulation of the secondary cell wall formation in conifers and its closest homolog in Arabidopsis is *AtMYB46* (Bomal et al., 2008). Transgenic spruces that overexpressed loblolly pine *PtMYB8* presented ectopic secondary cell wall deposition and an upregulation of CesA genes and other genes associated with cell organization and biogenesis (Bomal et al., 2008).

MYB transcription factors control the expression of target genes by interacting with specific DNA sequences in their promoter regions (Weirauch et al., 2014). Studies have shown that MYB transcription factors bind to cis-regulatory elements that are enriched in adenosine and cytosine residues, such as the AC-elements ACC[A/T]A[A/C][T/C] and ACC[A/T][A/C/T][A/C/T], and to the MYB-core sequences [C/T]NGTT[G/A] (Prouse and Campbell, 2012). Other transcription factors as AtMYB88 and AtMYB124 recognize a different consensus sequence, such as the [A/T/G][A/T/G]C[C/G][C/G] (Xie et al., 2010).

In this study, we investigated the developmental and tissue regulation of a white spruce *CesA3* gene to gain insight into its role in secondary xylem formation. Our specific objectives were to: (1) analyze the phylogeny of *CesA* gene sequences related to secondary cell wall formation in vascular development in conifers and angiosperms, (2) isolate a white spruce *CesA3* promoter and identify putative MYB regulatory binding sites, (3) analyze the tissue expression pattern of a *CesA3* promoter and GUS reporter gene fusion in transgenic white spruce plants, and (4) study promoter – transcription factor interactions by electrophoretic mobility shift assays (EMSA) of MYB recombinant proteins and cis-regulatory elements in the *PgCesA3* promoter.

## Materials and Methods

### Phylogenetic Tree

We searched for conifer CesA proteins that were related to secondary cell wall biosynthesis in the non-redundant protein NCBI database (nr) using Blastp, with an e-value threshold of 1e^-20^, and white spruce CesA1, CesA2, and CesA3 sequences as a query. We retained conifer CesA’s with at least a 75% amino acid sequence similarity and 90% sequence coverage with the white spruce CesA1, CesA2, and CesA3 proteins. Our phylogenetic analysis included only three white spruce (*P. glauca*) cellulose synthases, PgCesA1, PgCesA2, and PgCesA3 because the other CesA protein sequences were incomplete in the databases that we searched.

Angiosperm CesA proteins linked to primary and secondary cell wall formation were identified based on phmmer (*e*-value < 0.01),^1^ which provides more accurate detection of remote homologs than BLAST. We utilized the white spruce CesA1, CesA2, and CesA3 to search the UniProt database (The UniProt Consortium, 2015) for Arabidopsis (*Arabidopsis thaliana*), maize (*Zea mays*), rice (*Oryza sativa*), eucalyptus (*Eucalyptus grandis*), shinybark birch (*Betula luminifera*), poplar (*Populus trichocarpa*), and coniferales CesA proteins. We also verified in the literature that all of the published CesA proteins from these plant species were included in our sequence search results. See Supplementary Table S1 for accession numbers.

We aligned sequences using MAFFT version 7.0 and FTT-NS-I (iterative refinement method; 1000 iterations) strategies (Katoh and Standley, 2013). We constructed the phylogenetic tree using FastTree 2 (version 2.1.6, options: -boot 1000 -wag -gamma (Price et al., 2010). The tree was visualized with FigTree v1.4.2.^2^ Color modifications and group indications were made with Inkscape.^3^

### Gene Structure

To determine gene structure (introns and exons), we utilized the *CesA3* cDNA sequence (BT116976) and the genomic sequence containing the *CesA3* gene (BAC contig KC860240) published by Sena et al. (2014). The gene sequence was mapped onto the BAC contig sequence using est2genome incorporated in the annotation software MAKER (Cantarel et al., 2008). Gene structures from Arabidopsis, maize, poplar, and eucalyptus were recovered from the databases of TAIR 10^4^ and Phytozome (gene annotation of assembly v3 of *P. trichocarpa* and v2 of *E. grandis*; Tuskan et al., 2006; Goodstein et al., 2012; Myburg et al., 2014) and the Maize Genome Sequencing Project.^5^ We utilized Inkscape^6^ to build the figure.

### Promoter Analysis

The 5′-upstream sequence of the white spruce *CesA3* gene was obtained by genome walking (Genbank, KF824520) as described by Duval et al. (2014). The transcription start site was determined by 5′RACE as described by Bedon et al. (2009). The primers used for the identification and cloning of the *CesA3* gene 5′ genomic sequence was provided by Duval et al. (2014).

Putative regulatory MYB elements in the promoter sequence were identified using the Plant Transcription Factor Database, V4.0, and FIMO, a motif search tool (Grant et al., 2011). We downloaded the MYB transcription factor binding motifs from Arabidopsis and then screened the promoters of the *P. glauca CesA3* gene and its orthologs *AtCesA7* from Arabidopsis and *PttiCesA7-A* from *P. trichocarpa*. The promoter and 5′ UTR regions from *P. trichocarpa* and Arabidopsis were recovered from Phytozome 12^7^ (Potri.006G181900; 2000 bp upstream of the transcription start site) and the Eukaryotic Promoter Database^8^ (promoter ID AT5G17420_1; 2100 bp upstream of the transcription start site), respectively.

We searched for MYB TF motifs by using two different match threshold *p*-values, 0.001. and 0.0001, and compared the outcomes. We selected the best hit of each potential MYB binding motif, first with a threshold *p*-value of 0.001 and then 0.0001, for each species. With a threshold *p*-value of 0.001, we identified 66 MYB transcription factors that could bind the promoter region of the three species. As some hits were poorly conserved among the three species, we decided to be more stringent in our analysis, using a threshold *p*-value of 0.0001. Potential MYB binding sites in the *PgCesA3* promoter region were filtered and hits with a score superior to 31.9 were retained (see Figure 3). We aligned the promoter region sequence of *PgCesA3* and its orthologs in Arabidopsis and poplar and compared the best match of each TF binding motif between the three species.

### Transgenic Spruce Expressing the Ces3A Promoter-GUS Gene Fusion

A Pro*PgCesA3*::GUS (β-glucuronidase) fusion was created by inserting the putative promoter region into vector pMJM (Levée et al., 2009) as described in Bedon et al. (2009), digesting with Sbf1 and ligating it into the binary vector pCAMBIA2300.^9^ The binary vector was subsequently transformed into *Agrobacterium tumefaciens* strain C58pMP90 (Koncz and Schell, 1986). White spruce embryonal masses of line PG653 were transformed with Agrobacterium and selected with kanamycin as described in Klimaszewska et al. (2001). Following antibiotic selection to identify transgenic lines we identified 10 lines showing good embryonal masses growth. Six lines were selected randomly for plant production for further evaluation of GUS reporter gene expression. Plant production from individual transformed lines was as described in Klimaszewska et al. (2005) with the following modification: 3-month old somatic seedlings were produced by subculturing monthly and were acclimated in a growth chamber by removing the parafilm from the germination plates for the final 2–4 weeks of culture. Small transformed seedlings were evaluated for GUS reporter gene expression and all showed vascular specific expression. Three lines were selected for plant production and transferred to the greenhouse. Occasional hand misting of the plants was required for the first week after planting of the seedlings. Control lines (empty vector) were also selected for somatic embryo maturation and seedling production.

Somatic seedlings were planted in 3:1:1 mix of peat, vermiculite and perlite and grown in a greenhouse under a 16/8 h photoperiod with day/night temperatures of 24/20°C. At the end of the first growing season, when the plants had set a terminal bud, they were subjected to an accelerated growth schedule. Plants were hardened off by gradually reducing light and temperature conditions over a seven-week period to a final photoperiod of 8 h and day/night temperatures of 5°C. They were then subjected to a cold treatment by being put in a cold room at 2°C for six weeks after which they were returned to the greenhouse. Over an acclimatization period of two weeks, light and temperature were gradually increased from 8 h and 10°C to the original growing conditions. This accelerated growth schedule permits synchronous bud flush in all plants in the subsequent growth cycle.

Histochemical localization of GUS expression was evaluated in seedling tissues during both the first (data not shown) and second growth seasons. Seedling tissue was harvested from the three selected lines and subjected to GUS histochemical staining.

### Histochemical Staining of Plant Tissues

Expression analysis of the Pro*PgCesA3*::GUS (β-glucuronidase) fusion and sample preparation were adapted from Hawkins et al. (1997) as described by Bedon et al. (2009). Samples of roots, needles, stem just below the apical meristem, and first and second year stem segments were cross cut to 5–50 mm in length depending on the organ and pre-treated 30 min in cold 90% acetone (Hemerly et al., 1993). Samples were rinsed twice with 100 mM potassium phosphate buffer (pH 8), and incubated in 5-bromo-4-chloro-3-indoyl-β-D-glucuronic acid, with potassium ferricyanide and potassium ferrocyanide in sodium phosphate buffer (Vigneault et al., 2007) at 37°C in the dark under vacuum for 6–12 h until a blue stain was clearly visible. All tissue samples, except for stem, were then supported in 6% low melting temperature agar before cross-sectioning (30–50μm thickness) using a vibratome (Vibratome 1500, Vibratome Co., St. Louis, MO, United States). Sections were counterstained with phloroglucinol-HCl to visualize lignin deposition (Sibout et al., 2005). All tested lines showed the same patterns of GUS reporter tissue-specific expression.

### Expression and Purification of Recombinant MYB Proteins

Full-length white spruce MYB coding sequences (see accession numbers in Supplementary Table S2) were amplified by PCR with gene specific primers. The amplicons were cloned into the NdeI and XhoI restriction sites (New England Biolabs, Beverly, MA, United States) of the pET300/NT-DESTvector (ThermoFisher Scientific). The construct was confirmed by DNA sequencing and transformed into *E. coli* Rosetta^TM^ 2 (DE3) competent cells (Millipore Sigma). Bacterial growth and protein induction were performed at 37°C in LB broth containing kanamycin (30 μg/mL) and chloramphenicol (100 μg/mL). For protein induction, a bacterial culture at an O.D. 600 nm of 0.6 was used. Isopropyl-beta-D-thiogalacto-pyranoside (IPTG) was added to a concentration of 0.1–1.0 mM and the culture was incubated for 2–5 h for optimal production of each spruce MYB protein (see Supplementary Table S3). The cells were harvested by centrifugation (9000 × *g*, 10 min, 4°C), and pellets were resuspended in cold lysis buffer [20 mM Tris–HCl, pH 7.9, 100 mM NaCl, 5 mM imidazole plus one tablet of cOmplete, Mini, EDTA-free Protease Inhibitor Cocktail (Millipore Sigma)]. Recombinant proteins were purified by affinity chromatography using the HisBind Purification Kit (Millipore Sigma). Where protein recovery from inclusion bodies was necessary (PgMYB12 and 22), the inclusion bodies were denatured and solubilized in 5 ml denaturation buffer [8 M Urea, 20 mM Tris–HCl, pH 7.9, 500 mM NaCl, 5 mM imidazole plus one tablet of cOmplete^TM^, Mini EDTA-free Protease Inhibitor cocktail (Millipore Sigma)]. The cells were lysed using a Vibracell VC-50 sonicator and probe with a 3 mm micro tip. They were lysed on ice using 6 × 10 second bursts to prevent overheating of the samples. After sonication, the cells were incubated at room temperature for 1 h, with gentle rotation and then centrifuged at 10,000 × *g*, 30 min, 4°C. The supernatant was recovered and the recombinant proteins were purified by affinity chromatography using the His∙Bind^®^ Purification Kit (Millipore Sigma). The purified proteins were renatured by dialysis in renaturation buffer (20 mM Tris, 50 mM NaCl, 20 mM imidazole, pH 8.0) followed by dialysis buffer (20 mM Tris, 50 mM NaCl, pH 8.0) using Spectra-Por 1 dialysis membrane MWCO 6-8000 (Spectrum Labs). Protein purification was verified by SDS-PAGE analysis and Coomassie blue staining. The integrity of the purified recombinant proteins was evaluated by western-blot analyses using a mouse anti-His antibody (GE-Healthcare; Supplementary Figures S1–S5).

### Electrophoretic Mobility Shift Assay

Purified white spruce PgMYB5, PgMYB12, PgMYB13, and PgMYB22 recombinant proteins were tested for their ability to bind to oligonucleotides from the white spruce *CesA3* promoter using EMSA. We chose to test the sequence from region 32 (Supplementary Figure S6) of the *PgCesA3* promoter which contains two AC- like motifs in tandem. As the positive control, we used the canonical AC-I sequence from the *PAL* promoter gene (loblolly pine, accession number U39792.1; Patzlaff et al., 2003b). The 30bp fragments of the *CesA3* promoter used as probes were AC-I like, AC-I, mAC-I like and a fragment without a cis-element (Supplementary Table S4). The double-stranded oligonucleotides were end-labeled with γ-32P. 375 ng of recombinant protein and 100 ng of poly(dI-dC; used as a non-specific competitor) were incubated at room temperature for 30 min along with 0.2 ng labelled probe in a 20μl reaction. The incubation was done in binding buffer with a final concentration of 20 mM Tris pH 8.0, 10 mM NaCl, 2 mM EDTA, 2 mM dithiothreitol (DTT), and 10% glycerol (v/v). Competition reactions used unlabeled double-stranded probes in a 20 or 200-fold excess. Electrophoreses of the DNA–protein complexes were performed on a 6% native polyacrylamide gel at constant current of 35 mA (1 × Tris–glycine buffer, 4°C). Migration of radiolabeled probes and complexes were detected on Kodak Biomax XAR film (Bomal et al., 2014).

## Results

Three white spruce cellulose synthases (*PgCesA1, PgCesA2, PgCesA3*) that are preferentially expressed in secondary xylem were identified as candidate genes during the formation of secondary cell walls in vascular development (Raherison et al., 2012). Here, we presented sequence analyses along with functional studies of promoter and transcription factor binding to gain insight into their putative role and regulation.

### Three White Spruce CesA Genes Are Homologous to Secondary Cell Wall CesA Genes of Angiosperms

**FIGURE 1:**
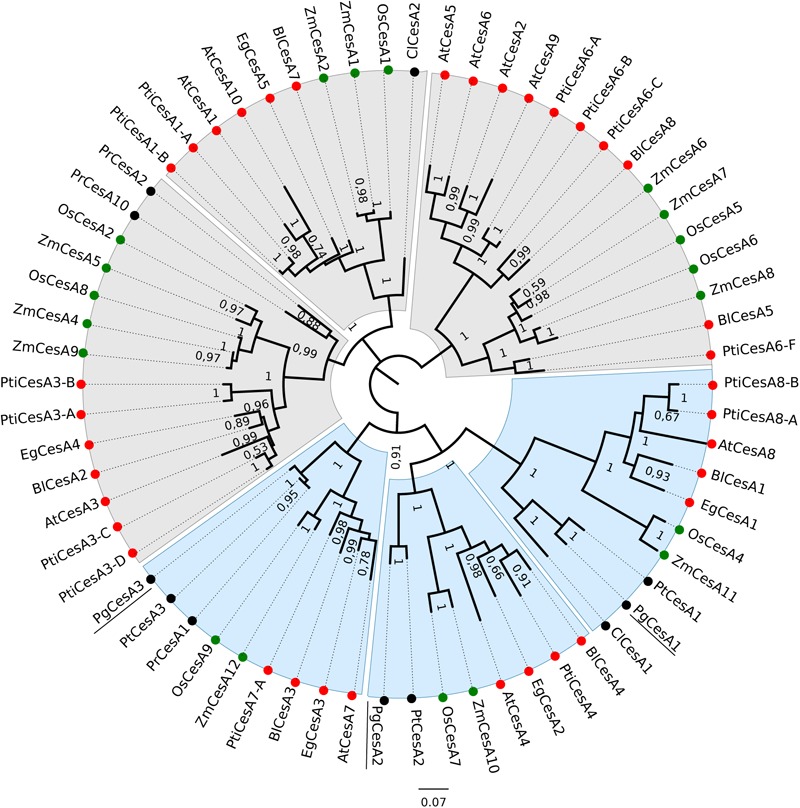
Phylogenetic tree of angiosperm and gymnosperm cellulose synthases. The unrooted tree was created with an alignment of 66 CesA protein sequences and FastTree. Clades containing CesA proteins mainly associated with primary cell wall synthesis are shaded in grey, and clades related to secondary cell wall biosynthesis are shaded in blue. Monocots are indicated with green circles, dicots with red circles and gymnosperms with black circles. Bootstrap values > 0.50 are indicated at branch points. The white spruce CesA genes are underlined. Species included in this analysis: Os: *Oryza sativa subsp. japonica*; Zm: *Zea mays*; At: *Arabidopsis thaliana*, Eg: *E. grandis*; Pti: *Populus trichocarpa*; Bl: *Betula luminifera*; Pr: *Pinus radiata*; Pt: *Pinus taeda*; Cl: *Cunninghamia lanceolate*; Pg: *Picea glauca*.

Our phylogenetic analysis of cellulose synthase sequences from white spruce, other conifers and angiosperms (monocots and dicots) clustered the CesA proteins into six main clades. Monocot, dicot and gymnosperm CesA sequences clustered into different subgroups within the six clades (Figure 1). Previous studies found similar groupings and reported that the members of the distinct clades were associated with either primary or secondary cell wall formation (Holland et al., 2000; Djerbi et al., 2005; Huang et al., 2014). The white spruce cellulose synthases grouped in clades with angiosperm cellulose synthases previously linked to secondary cell wall formation including AtCesA8, AtCesA4, and AtCesA7 from Arabidopsis, PtiCesA8, PtiCesA4, and PtiCesA7-A from poplar, and EgCesA1, EgCesA2, and EgCesA3 from Eucalyptus (Holland et al., 2000; Tanaka et al., 2003; Xi et al., 2017).

The three white spruce CesAs clustered together with three loblolly pine CesAs that were preferentially expressed in xylem tissue (Nairn and Haselkorn, 2005), suggesting conservation of expression and supporting our hypothesis that conifer CesA proteins may be functionally orthologous to angiosperm secondary cell wall CesA proteins.

In this study we focused on the functional role and characterization of the *PgCesA3* gene because of its potential involvement in cell-wall synthesis. Additionally, in our previous work (Duval et al., 2014) we isolated the *PgCesA3* promoter region and defined a set of potential transcription factors with the ability to regulate this given promoter.

### *CesA3* Gene Structure and Comparative Analysis With Angiosperms

**FIGURE 2:**
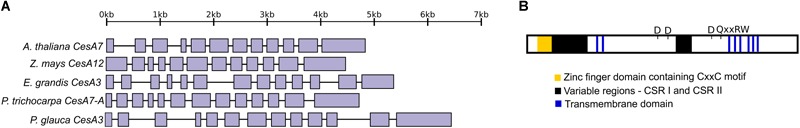
*CesA3* gene structure and protein domains. **(A)** Gene structure of the white spruce *CesA3* gene and its closest homologs in Arabidopsis, maize, eucalyptus, poplar and white spruce. Solid boxes represent exons and lines introns. **(B)** A diagrammatic representation of PgCesA3 protein. In yellow is indicated the zinc finger domain, in black the CSR I and CSR II and in blue the transmembrane domains. The approximative location of the processive glycosyltransferases motifs are indicated as D and QxxRW.

An analysis of gene structures showed a relatively conserved gene structure between the sequences from white spruce and angiosperms (Figure 2A). We analyzed the gene structure of *PgCesA3* by mapping the cDNA sequence (Raherison et al., 2012) on to the genomic sequence from a white spruce BAC containing the *CesA3* gene (Sena et al., 2014). We then compared the genomic sequence structure to those of the closest homologs from Arabidopsis (*AtCesA7*), maize (*ZmCesA12*), eucalyptus (*EgCesA3*) and poplar (*PtiCesA7-A*). The number of exons/introns varied slightly between species from 12 exons (11 introns) in maize and Arabidopsis to 13 exons (12 introns) in poplar, eucalyptus, and white spruce. The average exon length is similar between the four species, ranging from 240 bp in eucalyptus to 306 bp in white spruce. The total length of intronic sequences in the white spruce *PgCesA3* gene was 1.5 time (eucalyptus), two times (Arabidopsis and poplar) or three times (maize) greater than the angiosperms analyzed here.

We defined the PgCesA3 protein domains (Figure 2B; Supplementary Figure S7) as described in the literature (Richmond, 2000; Kumar and Turner, 2015; Maleki et al., 2016). As expected in CesA plant proteins, we identified the Zinc finger domain containing the “CxxC” motif, immediately after was the variable region, known as the class specific region (CSR I). There were two transmembrane domains near the N-terminal region and an other six toward the C-terminus. A second CSR was identified close to the middle of the protein. The signature motifs typical of the processive glycosyltransferases were present: motifs D (asparagine amino acid) and QxxRW (glutamine, arginine and tryptophan amino acids, respectively, as reviewed by Maleki et al. (2016).

Taken together the results of the comparative gene structure and the protein domains raise a question: is the occurrence of one more exon in the white spruce gene, compared to Arabidopsis, could be associated to a protein domain? To answer this question, we compared the exons encoding the protein domains between white spruce and Arabidopsis (as described by Richmond, 2000). We observed that the additional exon in white spruce harbors part of the CSR I region, which is longer in white spruce than in Arabidopsis. In Arabidopsis, as identified by Richmond (2000), the CSR I comprised the third and fourth exons while in white spruce it comprised the third to fifth exon. We aligned the white spruce CesA3 protein and the poplar homolog protein (PtiCesA7-A) and observed the same, poplar’s additional exons harbored part of the CSR I as well. The distribution of the other domains along the exons was similar among white spruce, Arabidopsis and poplar.

### *CesA3* Promoter Region Contains Several Potential MYB Binding Sites

**FIGURE 3:**
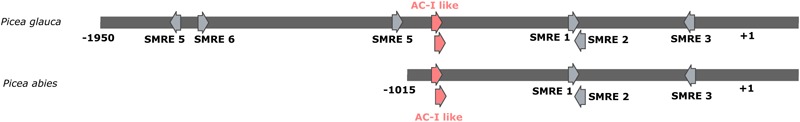
Map of the most relevant putative MYB binding sites in the upstream region of the white spruce and Norway spruce *CesA3* gene. The map with all putative MYB binding sites is in Supplementary Figure S6. The binding sites are identified by arrows. The region in red contains two AC-I like motifs, tested in the gel shift experiment from the present study. The transcription start site is indicated by +1.

We undertook an analysis of upstream sequences to gain insight into cis-regulation of *PgCesA3* gene expression. We isolated its putative promoter region immediately upstream of its coding sequence by using genome walking (see Methods for details). A genomic fragment was obtained that included a 167 bp 5′UTR and 1950 bp 5′ upstream of the putative transcription start site (+1). We also identified a Norway spruce scaffold containing a homologous *CesA3* gene and 5′ upstream sequences (Nystedt et al., 2013). Pairwise alignment showed that 99% nucleotide sequence identity was present between the two spruce sequences, which had an overlap of 167 bp of 5′-UTR and 1015 bp in 5′ upstream sequences; a 903 bp gap was observed in the Norway spruce sequence (Figure 3).

We identified 68 regions that harbor potential MYB binding sites in the *PgCesA3* promoter sequence by using a permissive threshold (*p*-value of 0.001) to facilitate the identification of motifs that are less conserved. We labeled putative binding sites for MYB transcription factors according to the unique identifiers in Arabidopsis binding site database (Plant Transcription Factor Database, V4.0; Supplementary Figure S6 and Supplementary Table S5). The regions labeled as 7, 8, 10, 11, 28, 29, 41, 42, 43, 44, 59, and 61 in the *PgCesA3* promoter (Supplementary Figure S6) contained five of the eight secondary wall MYB-responsive elements (SMRE), i.e., SMRE 1, 2, 3, 5, and 6, ACC(A/T)A(A/C)(T/C), described by Zhong and Ye (2012; Figure 3). The *PgCesA3* promoter lacked SMRE4, SMRE7, or SMRE8, that correspond to the sequence canonical elements AC-II, AC-III and AC-I, respectively (Figure 3). The other potential MYB binding sites were variations of the SMRE consensus sequence (Supplementary Table S6 and Supplementary Figure S6). The pairwise alignment with the available sequence of Norway spruce *CesA3* promoter showed the conservation of sequence and position of the SMRE elements (Figure 3). In comparison, we found the SMRE3 and SMRE5 elements in the *AtCesA7* Arabidopsis promoter region and, SMRE2, SMRE4, and SMRE5 in the poplar *PtiCesA7-A* promoter.

The potential binding sites included sites for MYB transcription factors previously found to trans-activate the white spruce *PgCesA3* promoter (Duval et al., 2014). These include: PtMYB8, a close homolog of AtMYB46/83 (regions in green and yellow; Supplementary Figure S6); PtMYB1, a close homolog of AtMYB20/43 (regions in black; Supplementary Figure S6); and PgMYB12, a close homolog of AtMYB15 (regions in blue; Supplementary Figure S6). We also identified putative binding sites for AtMYB103, which is involved in secondary cell wall biogenesis (Zhong et al., 2008).

Our analysis of potential MYB binding motifs identified 26 MYB transcription factors that can potentially bind motifs (threshold *p*-value of 0.0001) in the promoter region of both white spruce and Arabidopsis. Eight regions in the white spruce promoter and nine in the Arabidopsis promoter possess motifs for binding by these transcription factors (Supplementary Table S7). A comparison between poplar and white spruce revealed ten common MYB transcription factors that can potentially bind six and four regions, respectively (Supplementary Table S8). Among Arabidopsis, poplar and white spruce we identified eight MYBs in common (Supplementary Table S9), of which two were related to secondary cell wall formation, AtMYB83, and AtMYB43.

A higher level of conservation was found in the common potential MYB binding motifs between white spruce and Arabidopsis such as in SMRE 3 (ACCAAAC), than between white spruce and poplar where the best hit for AtMYB43 was an SMRE 5 (ACCTAAT) and in white spruce was an SMRE 2 (ACCAACT), both in the reverse strand. The common potential MYB binding motifs between poplar and white spruce did not present the exact same sequence. For example, the best hit in poplar for AtMYB43 was an SMRE 5 (ACCTAAT) and in white spruce it was an SMRE 2 (ACCAACT), both in the reverse strand. However, the majority of shared binding sites (best hits) between Arabidopsis and white spruce were SMRE 3 (ACCAAAC).

### *CesA3* Expression in Developing Xylem Tissues

**FIGURE 4:**
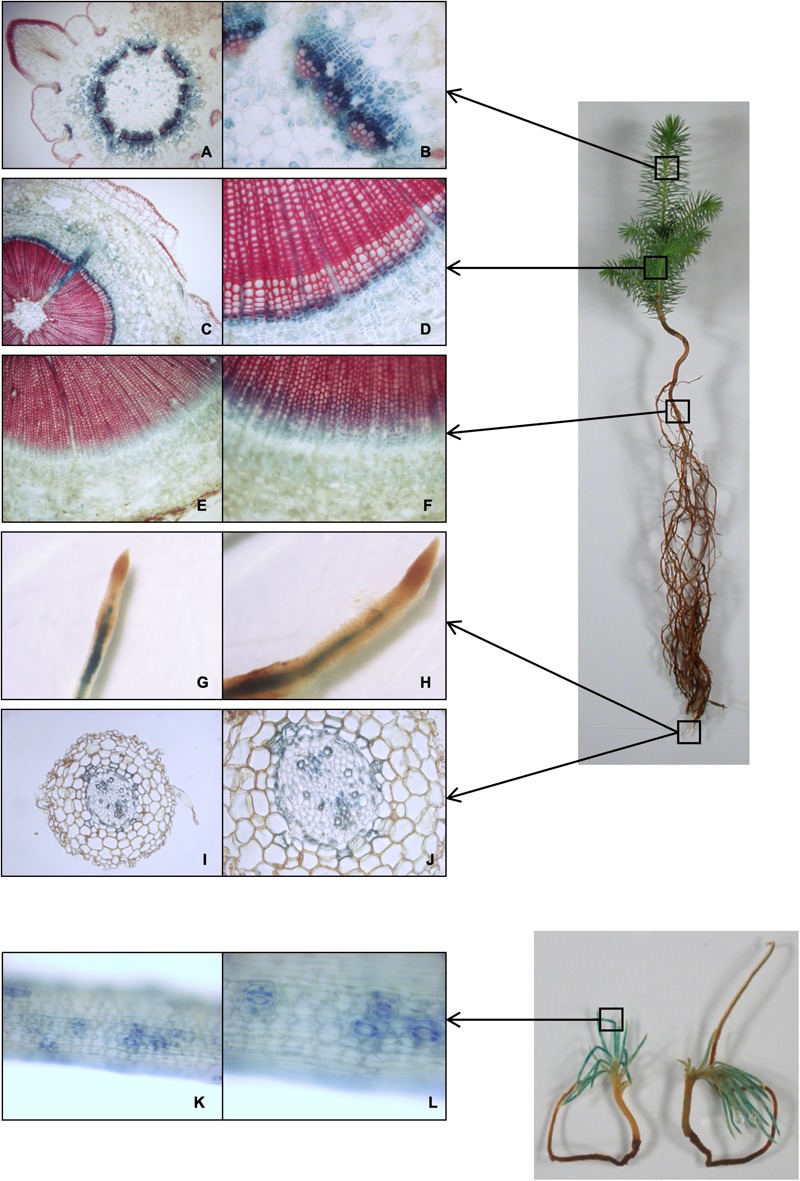
Histochemical localization of GUS activity driven by the *CesA3* gene promoter. Representative GUS staining in transgenic lines transformed with white spruce promoter CesA3::GUS construct. **(A,B)** cross section of first-year growth of the developing stem about ten days after bud flush; **(C,D)** cross section of second-year growth stem; **(E,F)** cross section of root; **(G–J)** root tip and cross section; **(K,L)** close-up view of a developing needle from a somatic embryo.

The *CesA3* promoter sequence was fused to the GUS reporter gene and used to stably transform white spruce (see Methods, for details). Histochemical staining of GUS activity driven by the putative *PgCesA3* promoter revealed intense blue staining in developing xylem tissues, close to the cambium zone (Figure 4). In the stem of 1^st^ and 2^nd^-year growth, we observed blue staining in the cytoplasm of differentiating xylem with no evidence of GUS staining in mature tracheids. The results were the same in roots but the blue staining was much lighter, resulting from a lower number of cell types harboring secondary cell wall development. Ray parenchyma cells also stained blue (Figure 4). Comparing the samples of the 2^nd^ year stem growth ten days after bud flush to the same tissue sampled just after budset we observed more cell rows of differentiating xylem in the latter (Supplementary Figure S8). Blue staining was also observed in both the xylem and the endodermis cells in root tips, and in the guard cells of developing needles from somatic embryos (Figure 4). These results are consistent with preferential expression in the developing xylem tissue in agreement with the pattern of the *PgCesA3* endogenous gene (Raherison et al., 2012).

### PgMYB12 Binds an AC-Like Sequence in the *PgCesA3* Promoter

Transient trans-activation assays revealed interactions between the *PgCesA3* promoter and several MYB transcription factors (Duval et al., 2014) based on simultaneous introduction of MYB-containing vectors and promoter-reporter gene constructs into spruce. Positive interactions were found with PgMYB12 but not with PgMYB5 and PgMYB13, both of which possess an LxLxL motif in their N-terminal region that can mediate transcriptional repression in plants (Bedon et al., 2010).

The analysis of the *PgCesA3* promoter identified several SMRE sequences, 1, 2, 3, 5 and 6, and variations of the SMRE consensus sequence but no AC elements (SMRE 4, 7, and 8). However, we identified AC- I like motifs (region 32 in Supplementary Figure S6 and Figure 3) and decided to investigate their interaction with PgMYBs by EMSA. Region 32 of the *PgCesA3* promoter contains two tandem AC-I like motifs, which have a single mismatch each compared to the canonical sequence of the AC-I element (Patzlaff et al., 2003a,b). In Norway spruce we observed exactly the same AC-I like motifs.

**FIGURE 5:**
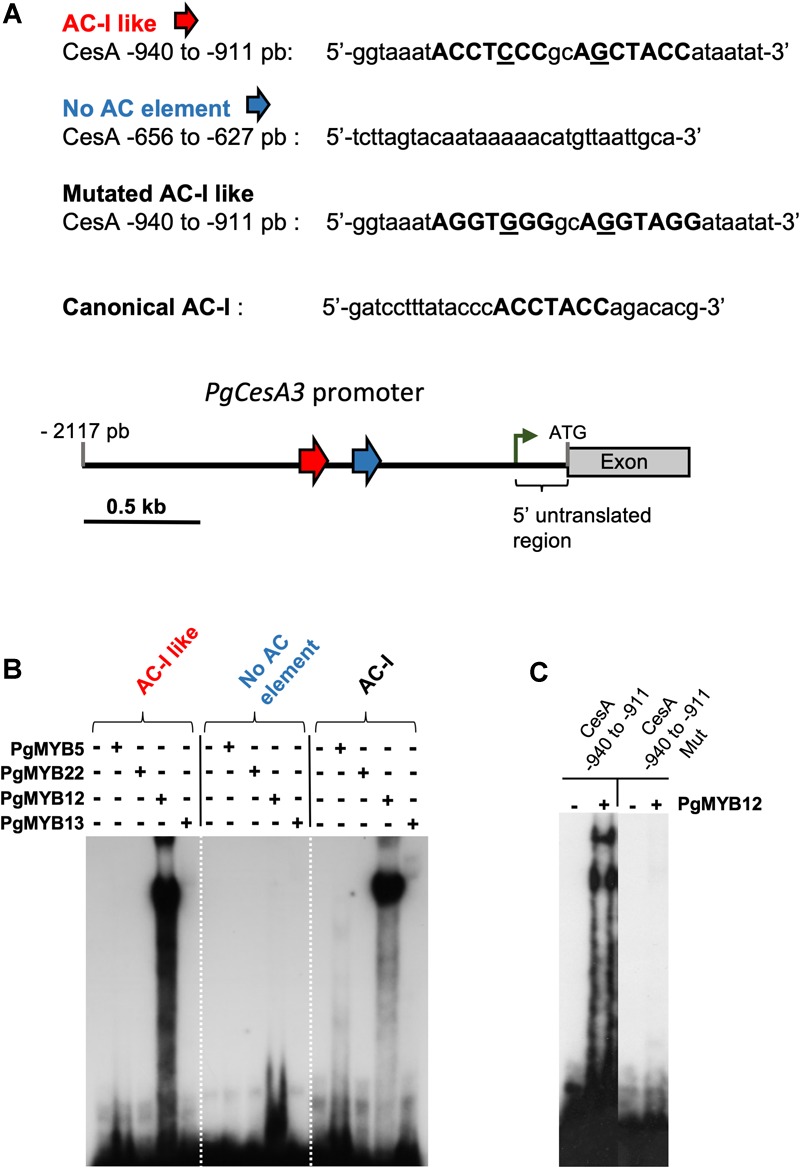
Electrophoretic mobility shift assay (EMSA) using different oligonucleotides probes from the white spruce CesA3 promoter. Purified white spruce PgMYB5, PgMYB12, PgMYB13 and PgMYB22 recombinant proteins were tested for their ability to bind labeled probes. **(A)** Nucleotide sequences of the different probes used with highlighted AC- rich elements. Distribution of the AC-I like elements are indicated by the red arrow and a control region without AC-I element in blue. The number on the left of the promoter indicates the relative distance to ATG. The green arrow indicates the position of the initiation of the transcriptional start site. **(B)** EMSA with the spruce MYB recombinant proteins and the four tested probes. **(C)** EMSA with PgMYB12 recombinant protein and the wild type or mutated CesA –940 to –911 probes.

According to the *in silico* analysis of the PgCesA3 presented in the previous section (3.3) demonstrating the absence of typical AC elements (I, II, and III) we put the emphasis of our study on the identified AC-I like element for further functional analyses. We used the available full-length spruce and pine sequences to produce recombinant MYB proteins (see Supplementary Table S2). We obtained EMSA compatible products for PgMYB5, PgMYB8 (Bomal et al., 2014), PgMYB12, PgMYB13 and PgMYB22 (see Supplementary Figures S1–S5 and Supplementary Tables S2–S3). The EMSA showed that PgMYB12 was able to bind to the AC-I like motif (region 32, Supplementary Figure S6) in the *PgCesA3* promoter, causing a mobility shift (Figure 5). PgMYB12 protein also bound to the AC-I canonical sequence, showing that it is not specific to just one binding sequence. The mutated probe of AC-I like did not induce band shifts with PgMYB12 (Figure 5). None of the five other MYBs were able to bind to either the AC-I element or the AC-like sequence (Figure 5).

## Discussion

### White Spruce *CesA1*, ***CesA2***, and ***CesA3 A***re Orthologous to Secondary Cell Wall CesA Proteins

We analyzed three white spruce cellulose synthases, *PgCesA1, PgCesA2, and PgCesA3*, that clustered in clades 4, 5, and 6 (Figure 1) with proteins known to be involved in secondary cell wall formation in angiosperms. The assembly of a functional secondary cell wall cellulose synthase complex requires three protein subunits in Arabidopsis (*AtCesA8/4/7*; Taylor et al., 2003; Hill et al., 2014) and rice (*OsCesA4/7/9*; Tanaka et al., 2003) and these different protein subunits also cluster in the clades 4, 5, and 6). The white spruce CesA proteins were orthologous to the three CesA subunit triplets in Arabidopsis, rice, eucalyptus, shinybark birch and the conifer loblolly pine. This observation suggests that there is a one-to-one orthology of the triple subunits among angiosperms and gymnosperms, which are likely to have derived from gene duplications before the split of angiosperms and gymnosperms (330 myr; Savard et al., 1994). In contrast, triplets of secondary cell wall CesA subunits were not found in the conifers Monterey pine (*Pinus radiata*) and China fir (*Cunninghamia lanceolata*), which could be due to gene loss in those species or deeper gene sampling in white spruce (Rigault et al., 2011) and loblolly pine (Nairn and Haselkorn, 2005). Poplar is the only species which presented an additional copy (CesA8 in clade 6), probably as the result of the recent whole genome duplication in the Salicaceae (Tuskan et al., 2006; Takata and Taniguchi, 2015).

The xylem preferential expression of the white spruce CesA genes (Raherison et al., 2012) supports a role in secondary cell wall formation and suggests they may be functional orthologs to CesA genes from other plant species. For example, Arabidopsis *CesA4/7/8*, eucalyptus *CesA1/2/3*, maize *CesA10/11/12*, poplar *CesA4/7-A/8-A/8-B*, shinybark birch *CesA1/3/4* and loblolly pine *CesA1/2/3* all presented high expression in stem or stalk, tissues that undergo secondary cell wall synthesis in xylem cells (Appenzeller et al., 2004; Hamann et al., 2004; Nairn and Haselkorn, 2005; Ranik and Myburg, 2006; Suzuki et al., 2006; Huang et al., 2014).

The additional exon in white spruce and poplar harbored part of the CSR I region which is longer in these two species than in Arabidopsis. The CSR is variable among species and is hypothesized to be involved in the formation of the rosette-like cellulose synthase complexes, helping to stabilize the CesA complex assembly (Sethaphong et al., 2013). It is unknown if the additional exon in perennial tree species has an impact on expression or protein function; however, phylogenetic inferences together with the expression profiles suggest conservation of function among proteins related to secondary cell wall biosynthesis.

### Spruce *CesA3* Promoter Contains MYB Binding Sequences

We analyzed the promoter region of the spruce *PgCesA3* gene and identified several putative MYB cis-regulatory elements including the SMRE 1, 2, 3, 5, 6 (Figure 3) and variations of the SMRE consensus sequences (Supplementary Figure S6). The variety of MYB binding sites could have a direct impact on gene regulation. It was shown that the eight SMRE variants have different responses to activation by AtMYB46/83 (Zhong and Ye, 2012). The same was observed in poplar, eucalyptus and pine orthologs where poplar PtrMYB2/3/20/21, eucalyptus EgMYB2 and pine PtMYB4, which were shown to differentially bind to and activate the eight variants of SMRE consensus sequences (Zhong et al., 2013). We noted that many different MYBs have the potential to bind to the same regions (Supplementary Figure S6 and Supplementary Table S5). The motifs that contain an SMRE can potentially bind several MYB proteins involved in the regulation of secondary wall biosynthesis genes (Zhong and Ye, 2014) as was shown for AtMYB46, AtMYB83, AtMYB43, AtMYB103, as well as MYBs AtMYB15, AtMYB62, AtMYB99, AtMYB116. Zhong and Ye (2012) showed that AtMYB46 and its paralog AtMYB83 bound to all eight variants of the SMRE consensus sequence ACC(A/T)A(A/C)(T/C). ATMYB58 and ATMYB63 are lignin-specific activators that may bind to three variants of SMRE that are identical to the AC-elements (Zhou et al., 2009), which also bound by AtMYB15 involved in wound response (Chen et al., 2006). There is considerable overlap among MYB DNA-binding sequences, probably because the R2R3 MYB DNA-binding domains are highly conserved (Stracke et al., 2001). Soler et al. (2015) showed that R2R3–MYBs controlling lignin and secondary cell wall biosynthesis were conserved across Arabidopsis, eucalyptus, poplar, grape, and rice. In eukaryotes, the overall nucleotide diversity in the promoter region is much higher than in the DNA coding sequence; however, cis-regulatory elements are conserved among orthologous genes (Lockton and Gaut, 2005). We compared the CesA promoter regions of Arabidopsis, poplar, and spruce and identified shared MYB binding sites for AtMYB43, AtMYB46, AtMYB83, and AtMYB103, which are involved in the regulation of secondary cell wall. This conservation of putative MYB cis-regulatory elements suggests that transcriptional regulation of cellulose biosynthesis appeared early in the evolution in vascular plants. We found a higher level of conservation in MYB DNA binding sites between white spruce and Arabidopsis than between white spruce and poplar. The time of divergence between gymnosperms and angiosperms is approximately 330 myr (Savard et al., 1994) and the time of divergence between poplar (Fabid) and Arabidopsis (Malvid) is approximately 82 myr (Clarke et al., 2011). This pattern may result from the slower rates of evolution in conifers than angiosperms (Buschiazzo et al., 2012) and a higher rate due to whole genome duplications in poplar and Arabidopsis, amongst other reasons. It has been shown that duplicated CesA genes in poplar diverged in a few regulatory elements, possibly causing differential expression (Takata and Taniguchi, 2015).

### *CesA3* Promoter in White Spruce Is Linked to Secondary Cell Wall Biosynthesis

The *PgCesA3* promoter was strongly activated in differentiating stem xylem cells according to GUS expression of the white spruce *CesA3* promoter fused to the β-glucuronidase reporter gene. Our results suggest that *PgCesA3* is involved in the deposition of the secondary cell wall during xylogenesis. One of the phases of xylem differentiation is the deposition of a thick multilayered secondary cell wall, which is coordinated and driven by numerous proteins, including cellulose synthases (Plomion et al., 2001). Some angiosperm CesA genes have been reported for their roles in the biosynthesis of the secondary cell wall. For example, the eucalyptus promoter *EgCesA3* exhibited GUS staining in Arabidopsis tissues undergoing secondary cell wall deposition (Creux et al., 2008). The poplar *PtiCesA7-A* gene was implicated in the multilaminar structure of the secondary cell wall (Xi et al., 2017) and the Arabidopsis *AtCesA7* was reported as essential for cellulose biosynthesis in secondary cell walls (Taylor et al., 1999, 2000).

In the present study, we observed that the promoter of *PgCesA3* drove xylem preferential GUS expression most strongly in differentiating xylem cells of developing stem and produced lighter blue staining in needles and roots. Our observations are consistent with microarray data showing that *PgCesA3* transcripts were most abundant in xylem tissue and detected at lower levels in foliage and roots (Raherison et al., 2012). We found that the *PgCesA3* promoter was also activated in the stomata guard cells. During stomatal movements, the cellulose microfibrils in the stomata undergo dynamic reorganization to enable aperture and closure driven by changes in turgor pressure. An Arabidopsis secondary cell wall *AtCesA7* mutant had smaller stomatal opening and smaller guard cells (Liang et al., 2010); interestingly, the guard cells were not deficient in cellulose, but displayed a loss of cellulose reorganization during stomatal movement. It is unknown if these mutant characteristics were due to collapsed xylem and compromised water transport or to the loss of cellulose reorganization (Rui and Anderson, 2016). Together, these observations suggest that *PgCesA3* could be necessary for stomatal function but additional studies will be needed to elucidate its specific role.

### PgMYB12 Interacts With AC-I Like Motifs in the *PgCesA3* Promoter

The spruce *PgCesA3* promoter and its putative ortholog in Arabidopsis both lack AC-I elements corresponding to SMRE4, SMRE7, and SMRE8. The poplar ortholog also lacks SMRE7 and SMRE8 but has SMRE4 elements. Due to the absence of AC-I elements, we hypothesized that an AC-I like element may function as a MYB binding site in the *PgCesA3* promoter.

Here, we found that PgMYB12 bound to both a canonical AC-I element (from PAL promoter, Patzlaff et al., 2003b) and also a region in the *PgCesA3* promoter containing two AC-I like elements. Previous work showed that PgMYB12 transactivated the expression of cellulose synthase, *PgCesA3* using an embryogeneic cell culture (Duval et al., 2014). PgMYB12 is a close homolog to AtMYB15 in Arabidopsis, which binds to AC-I elements in shikimate pathway genes (Chen et al., 2006). PgMYB12 could be involved in the regulation of cellulose biosynthesis in spruce and could regulate *PgCesA3* in roots based on it is preferential expression in root xylem of young and mature spruce trees (Bedon et al., 2007), which is in agreement with our GUS expression results (Figure 4).

It was also shown that neither PgMYB5 nor PgMYB13 were able to activate *PgCesA3* expression (Duval et al., 2014). Both MYBs are part of subgroup 4 and present an EAR core motif (Bedon et al., 2010), suggesting they could mediate transcriptional repression of specific genes. We found that they did not bind to the AC-I canonical sequence and the AC-I Iike motif. They could potentially bind to other elements in the *CesA3* promoter, competing with cognate transcription factors or sequestrate components of the transcriptional machinery away from the cis-regulatory DNA elements (Gill and Ptashne, 1988), since they present the basic helix-loop-helix (bHLH) interaction site (Zimmermann et al., 2004). Duval et al. (2014) showed that PgMYB22 was able to trans-activate the *PgCesA3* promoter as well as the promoters of genes related to the phenylpropanoid (*CAD, PAL*) and shikimate pathways (*DAHP*), and also those of transcription factors (*PgMYB1, PgMYB8*, and *PgLIM1*). In the present study we have not been able to identify specific DNA-protein interactions using EMSA for PgMYB22 nor for PgMYB8 or PgMYB15 (Figure 5).

In Arabidopsis, AtMYB46 binds directly to the promoters of *AtCesA4, AtCesA7*, and *AtCesA8* involved in secondary cell wall formation in *in vivo* and *in vitro* studies (Kim et al., 2012) and may directly regulate their expression (Ko et al., 2009). The closest ortholog to AtMYB46 in white spruce and in loblolly pine is PgMYB8 and PtMYB8, respectively (Bedon et al., 2007). As PtMYB8 transactivated white spruce *PgCesA3*, it is highly likely that Pt/PgMYB8 are functionally related to AtMYB46 and that the regulation of CesA genes involved in the secondary cell wall synthesis is conserved among angiosperms and conifers (Duval et al., 2014). Testing this hypothesis will be the next step in the understanding of the regulation of spruce cellulose synthase genes.

The affinity of conifer R2R3-MYBs for AC elements has been reported in pine, PtMYB1 (Patzlaff et al., 2003b) and PtMYB4 (Gómez-Maldonado et al., 2004; Patzlaff et al., 2003a); in maritime pine, PpMYB8 (Craven-Bartle et al., 2013) and in spruce, PgMYB8, PgMYB14 and PgMYB15 (Bomal et al., 2014). However, the interaction of these MYBs with potential DNA MYB binding sites in the promoter of a conifer cellulose synthase had not been tested before the present study. The potential MYB binding sites identified in this work open avenues for examining the interaction between other PgMYBs and the *PgCesA3* promoter motifs. (Duval et al., 2014).

## Author Contributions

All authors contributed to the conception and design of the experiments JS performed the bioinformatic analyses and drafted the manuscript. DL produced the transgenic plants and performed the histochemical analyses. ID planned the recombinant proteins and EMSA experiments and supervised the laboratory work. TN and DS purified recombinant proteins and performed the EMSAs. AS contributed to the supervision and discussion of the research. AS and JM revised the manuscript.

## Conflict of Interest Statement

The authors declare that the research was conducted in the absence of any commercial or financial relationships that could be construed as a potential conflict of interest.

## Footnotes

1 https://www.ebi.ac.uk

2 http://tree.bio.ed.ac.uk

3 https://inkscape.org

4 http://arabidopsis.org

5 http://www.maizesequence.org

6 https://inkscape.org

7 https://phytozome.jgi.doe.gov

8 http://epd.vital-it.ch

9 https://cambia.org/
