# Supplementary material for: Functional Analysis of the PgCesA3 White Spruce Cellulose Synthase Gene Promoter in Secondary Xylem
Source: Front Plant Sci. 2019 May 28;10:626. doi: 10.3389/fpls.2019.00626 (PMC6546725; doi:10.3389/fpls.2019.00626)
Supplement: Supplementary file 1 [file Data_Sheet_1.PDF]

Supplementary Material

1.1 Supplementary Figures

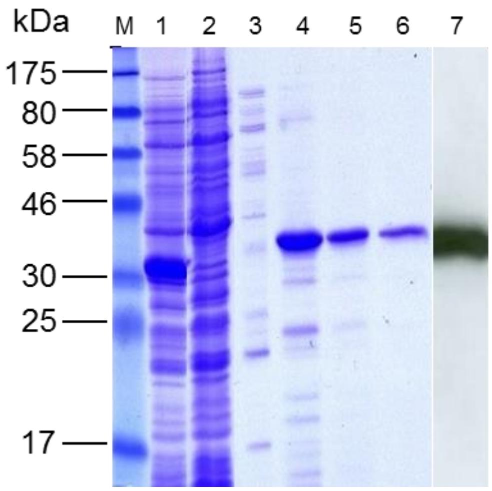

Supplementary Figure 1. SDS-PAGE and Western blot for PgMYB5. M, proteins marker; lane 1, total proteins from induced cells; lane 2, flow through fraction 1; lane 3, washing fraction with 60 mM imidazole; lane 4, elution fraction 1; lane 5, elution fraction 2; lane 6, elution fraction 3; lane 7, purified His tagged PgMYB5 (30.8 kDa) visualized by Western blot using a mouse anti-His antibody.

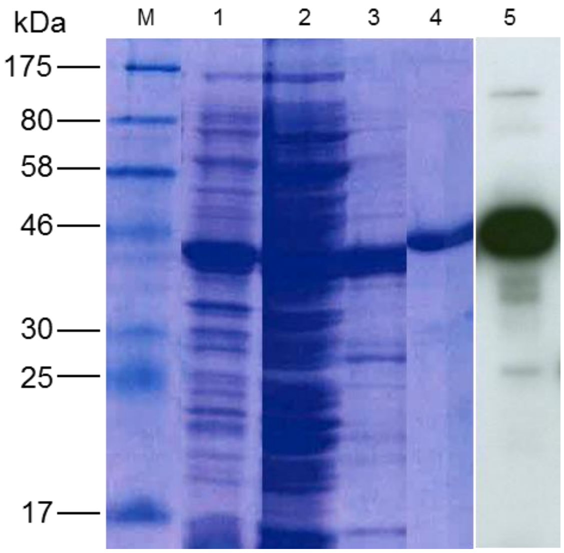

Supplementary Figure 2. SDS-PAGE and Western blot for PgMYB12. M, proteins marker; lane 1, total proteins from induced cells; lane 2, flow through fraction 1; lane 3, washing fraction with 60 mM imidazole; lane 4, elution fraction; lane 5, purified His tagged PgMYB12 (43.2 kDa visualized by Western blot using a mouse anti-His antibody.

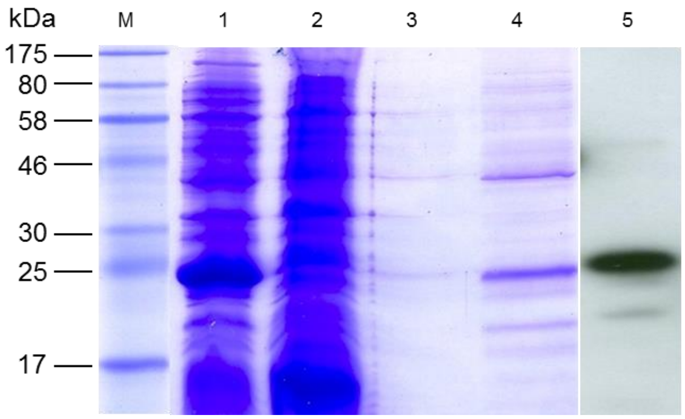

Supplementary Figure 3. SDS-PAGE and Western blot for PgMYB13. M, proteins marker; lane 1, total proteins from induced cells; lane 2, flow through fraction; lane 3, washing fraction with 80 mM imidazole; lane 4, elution fraction; lane 5, purified His tagged PgMYB13 (24.5 kDa) visualized by Western blot.

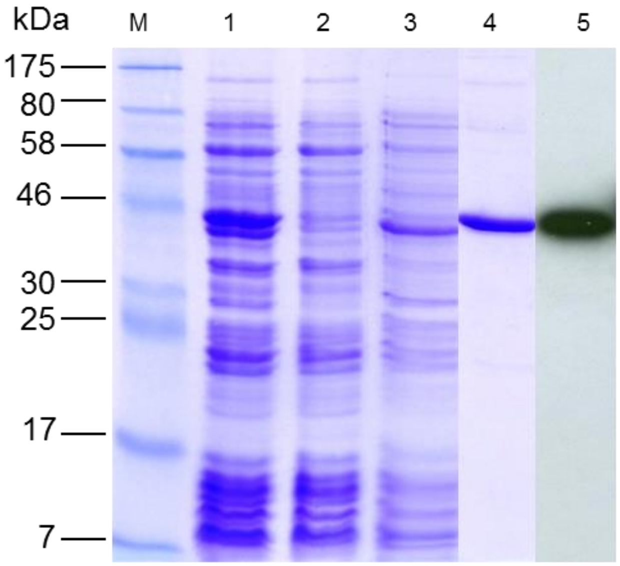

Supplementary Figure 4. SDS-PAGE and Western blot for PgMYB22. M, proteins marker; lane 1, total proteins from induced cells; lane 2, flow through fraction; lane 3, washing fraction with 60 mM imidazole; lane 4, elution fraction; lane 5, purified His tagged PgMYB22 (42.6 kDa) visualized by Western blot using a mouse anti-His antibody .

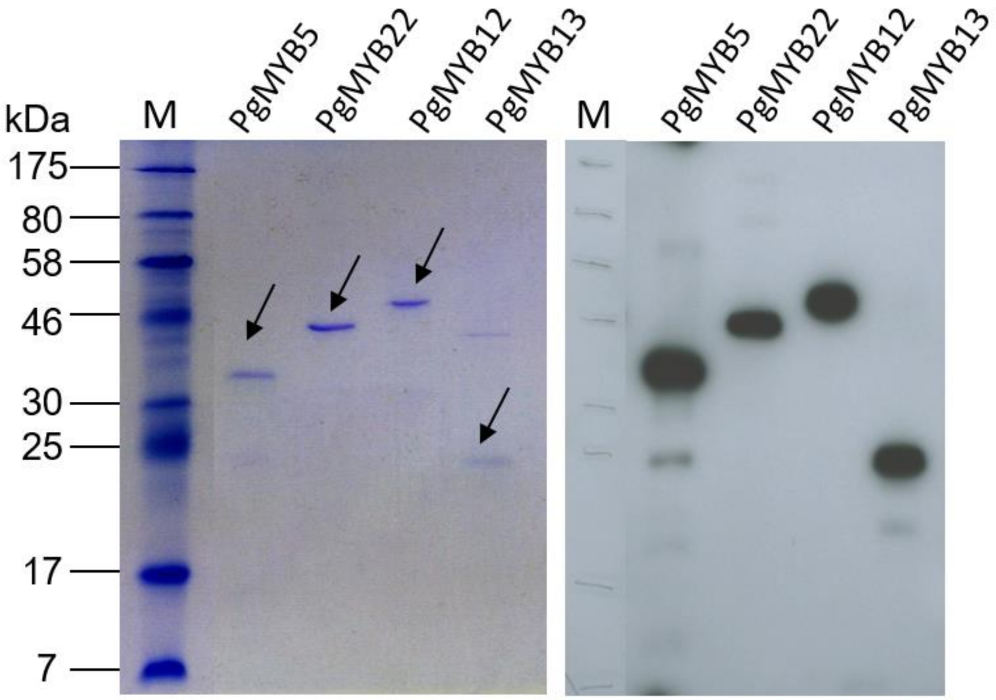

Supplementary Figure 5. SDS-PAGE and Western blot for all recombinant MYB proteins. M, proteins marker. Arrows on the left panel indicate the positions of the recombinant proteins after Coomassie blue staining of the polyacrylamide gel. The right panel shows recombinant His tagged MYB proteins visualized by Western blot using a mouse anti-His antibody.

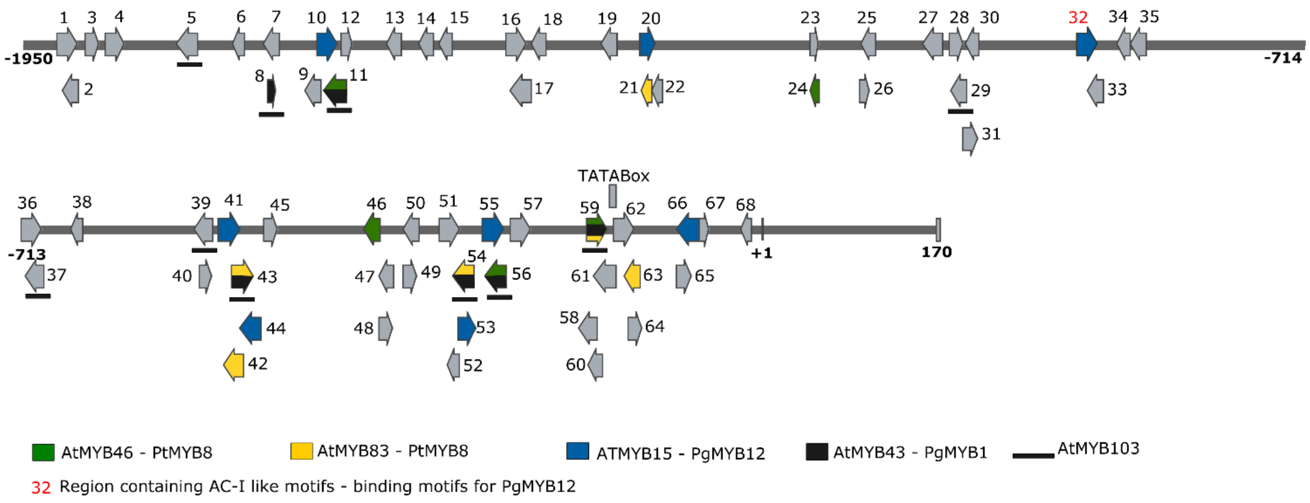

Supplementary Figure 6. Map of putative MYB binding sites in the upstream region of the *PgCesA3* gene. The binding sites are identified by numbered arrows. The sequence motifs, SMRE type, position and MYBs that may bind to each region are listed in Supplementary table S4. Green, yellow, black and underlined arrows represent motifs that may be bound by MYBs implicated in the regulation of secondary cell wall formation genes. The region 32 in red contains two AC-I like motifs, tested in the gel shift experiment from the present study. The transcription start site is indicated by +1.

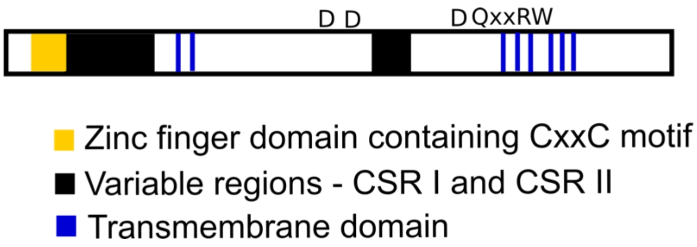

MEASAGLVAGSHNRNEFVVIHGHEEPKPLNSLSGHV**C**QI**C**GEDVGLNTDGELFVA**C**NE**C**GF
PV**C**RP**C**YEYERREGNQS**C**PQ**C**NTRYKRHRGSP**RVEGDDDEEDVDDIEQEFNMETQQRNR**
QQITEAMLHGRMSYGRGPDDENSQIAHNPELPPQIPILANGHSVVSGEIPTSYYAENQLL
ANPAMLKRVHPSSEPGSGRIIMDPNRDIGSYGFGNVAWKERADGYKSKDNKSGQLDM
TEGRYQYNGGFAPNEPEDYIDPDLPMTDEARQPLSRKVPIPSSKINPYRMVIVIRLIVLAIFL
RYRLLNPVKNAYGLRGTSIVCEIWFALSWILDQFPKWFPISRETYLDRLALRYEREGEPSMLA
SVDLFVSTVDPLKEPPLVTANTVLSILSVDYPVDKVSCYVSDDGASMLTFESLSETSEFARK
WVPFCKKFSIEPRAPEIYFSQKIDYLKDKFQPTFVKERRAMKREYEEFKVRINRMVAKASKV
PKEGWTMQDGTPWPGNNTRDHPGMIQVFLGHSGGLDTDGNELPRLVYVSREKRPGFQHHK
KAGAMNALVRVSAVLTNAPFMLNLDCDHYINNSKAIREAMCFMMDPQVGRKVCYVQFPQ
RFDGIDRNDRYANRNTVFFDINMKGLDGIQGPVYVGTGCMFRRQALYGY**GPPKGPKRPK**
MVTCDCLPCCGPRKKLNKSPKKTSGKKSAGIPAPAFNLDGIEEGVEGYDDERALLMSQ
LDFEKKFGQSSAFVQSTLMENGGVPQTANPAELLKEAIHVISCGYEDKTEWGKELGWIYGS
VTEDILTGFKMHSRGWRSIYCMPKRAAFKGSAPINLSDRLNQVLRWALGSVEIFMSRHCPL
WYGYGGGLKWLERFAYINTIVYPLTSLPLIAYCTLPAVSLLTGKFVIPQISTFASLFFIALFISIF
ATGILEMRWSGVSIEEWWRNEQFWVIGGVSAHFFAVIQGLLKVLAGIDTNFTVTAKASDDG
EFGELYAFKWTTLLIPPTTLLVINLVGVVVGVADAINNGFQSWGPLLGKLFFAFWVIVHLYP
FLKGLMGKQNRTPTIVVIWSILLASVFSLFWVRIDPFLSKVKGPDTKQCGINC

Supplementary Figure 7. Schematic organization of the PgCesA3 protein.

A

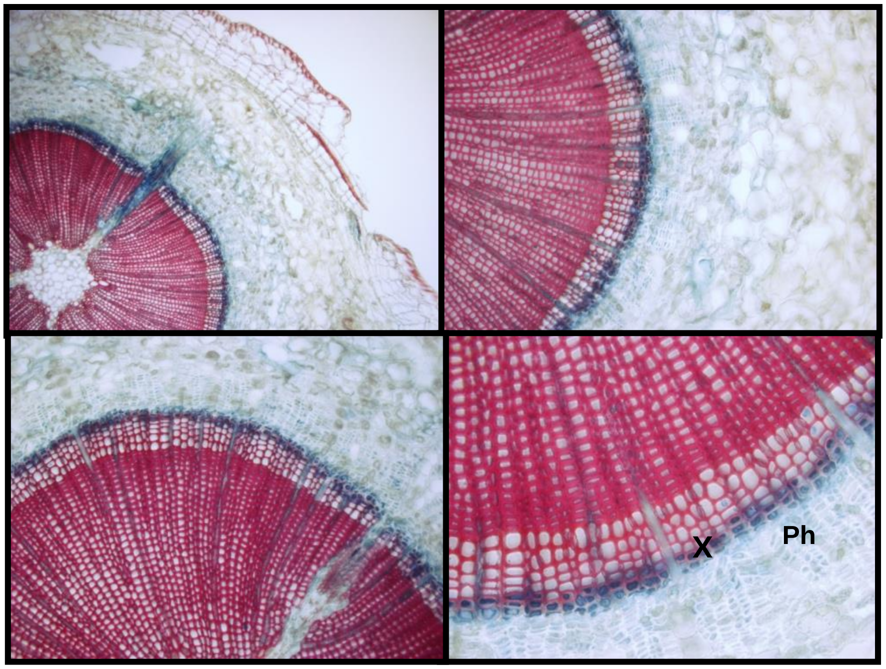

B

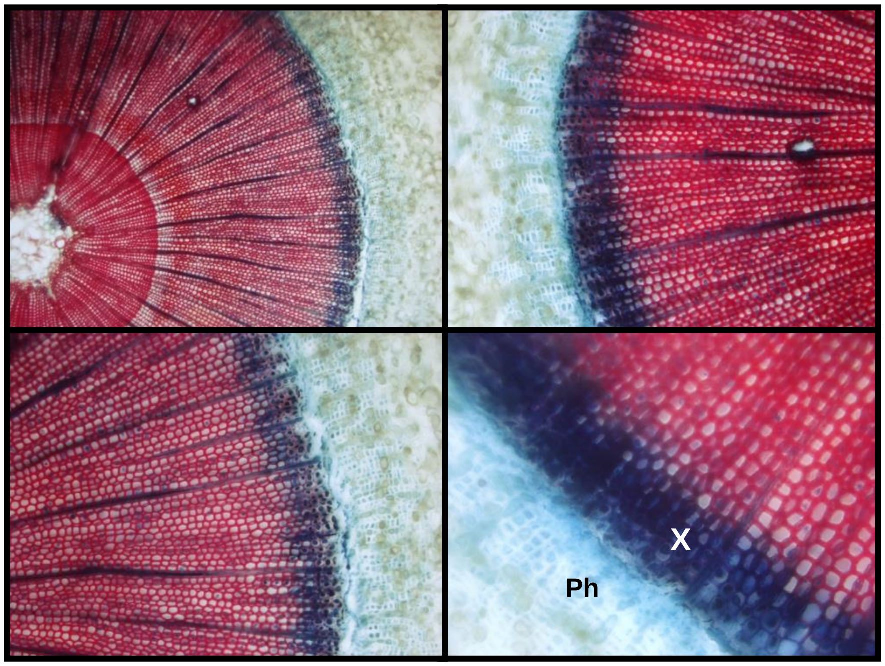

Supplementary Figure 8. Samples of the second-year growth stem. A) Stem, second year growth, approximately ten days after bud flush. B) Stem, second year growth, just after bud set.

X: developing xylem, Ph: Phloem.
